# Tenosynovial giant cell tumours of the upper and lower cervical spine: two case reports

**DOI:** 10.1038/s41394-022-00538-2

**Published:** 2022-08-03

**Authors:** Ning Zhu, Robert Campbell, Ananthababu Pattavilakom Sadasivan

**Affiliations:** 1 Department of Neurosurgery, Princess Alexandra Hospital, Brisbane, QLD Australia; 2 Department of Neurosurgery, Mater Hospital, Brisbane, QLD Australia; 3 Department of Neurosurgery, Queensland Children’s Hospital, Brisbane, QLD Australia

**Keywords:** Spinal cord diseases, Spinal cord

## Abstract

**Introduction:**

Tenosynovial giant cell tumours (TSGCTs) usually arise from the synovial membranes of tendon sheaths, bursa, and joints. They are rarely found in the spine. Lesions of the upper cervical spine (C1/2) are extremely rare, with only 13 previous cases reported in the literature. Of these, all previous anterior upper cervical cases (C1/2) have been deemed unresectable and have been managed with immunotherapy or radiological surveillance.

**Case presentation:**

We report two cases of TSGCST in the cervical spine: one with a lesion at C1/2 and another at C6/7.

**Discussion:**

The location of our C1/2 lesion was unique, allowing for a new endoscopic endonasal tissue biopsy method and a new transoral surgical approach for successful gross total resection. Our C6/7 lesion had a more typical location and was removed via a C6/7 laminectomy.

## Introduction

Tenosynovial giant cell tumours (TSGCTs) are benign but locally aggressive primary fibrohistiocytic tumours that usually arise from the synovial membranes of tendon sheaths, bursa and joints [1, 2]. They generally occur in large load bearing joints such as the hips and knees [1, 3]. They are rarely found in the spine but, when present, are generally found in the lower cervical and lumbar regions [4]. Lesions of the upper cervical spine (C1/2) are extremely rare, with only 13 cases reported in the literature [5]. Gross total resection is recommended for all TSGCTs due to their locally aggressive nature and the subsequent risk of joint instability from bone erosion and of neurological deficit from spinal cord compression [1, 2, 5–7]. However, in recurrent or inoperable cases, immunotherapy, radiation therapy and radiological surveillance are used [2, 4, 8]. 9 of the 13 previous upper cervical cases underwent gross total resection, while 3 were managed conservatively with serial MRI scans and 1 underwent immunotherapy [2, 3, 5–14]. This case report includes two patients with TSGCSTs in the cervical spine: one with a lesion between C1/2 and another at C6/7. Our first case (C1/2) describes a unique tumour location as well as a new endoscopic endonasal tissue biopsy method and a new transoral surgical approach which allowed for a successful gross total resection. Previous tumours with a similar anterior upper cervical location were deemed inoperable and were either managed with radiological surveillance or immunotherapy [11, 14]. We hope that this paper can add to the current literature to improve understanding, diagnosis, and management of future cases of TSGCTs.

## Case presentation

### Case 1 - C1/2 localized type TSGCT

**Fig. 1:**
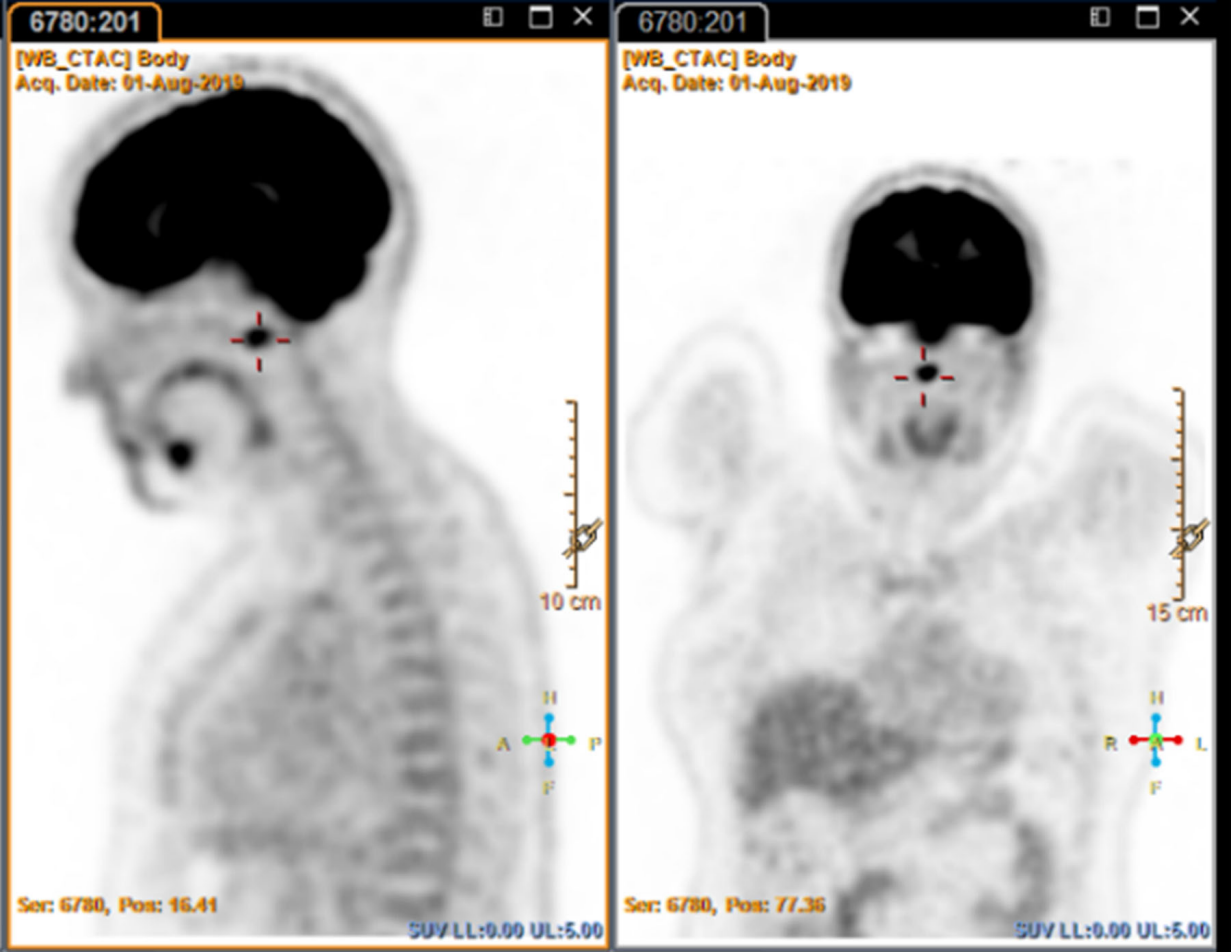
Case 1 PET scan. Moderate to intense FDG uptake in the upper cervical spine between the clivus and the anterior arch of C1.

A 48-year-old woman presented with a 3-year history of persistent right sided neck pain. This was associated with reduced neck and jaw range of motion and mild proximal bilateral upper limb weakness. Given her recent diagnosis of cervical cancer, she underwent a positron emission tomography (PET)/computed tomography (CT) scan to exclude metastases. PET scan showed moderate to intense fluorodeoxyglucose (FDG) uptake in the upper cervical spine between the clivus and the anterior arch of C1. See Fig. 1.

**Fig. 2:**
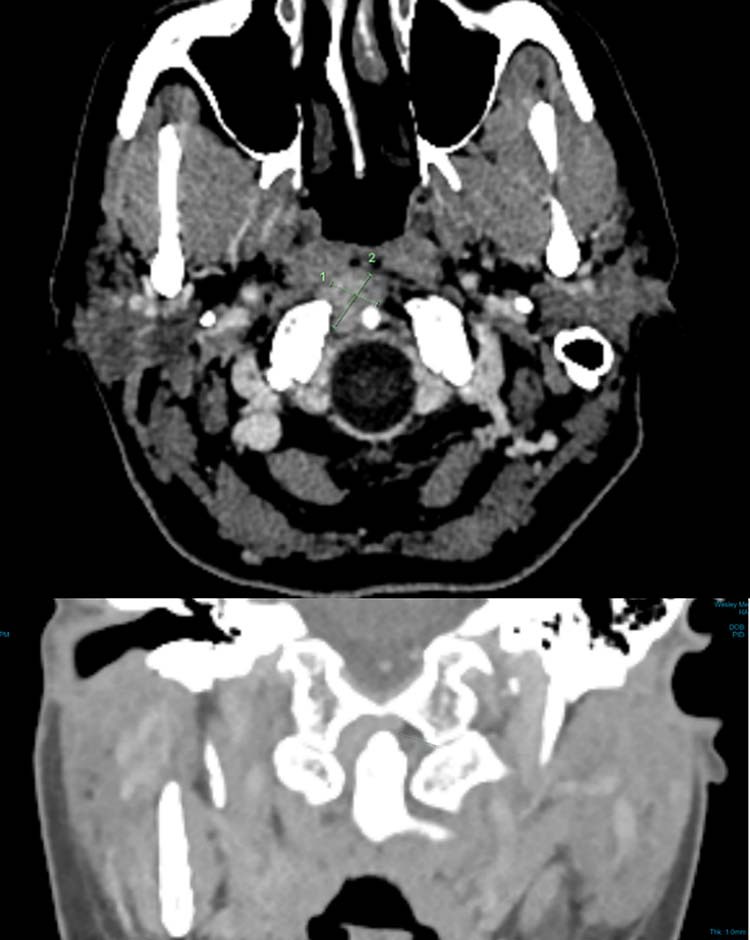
Case 1 CT scan. There was a soft tissue density, measuring 15 x 12 mm (AP x W), superior to the right side of the anterior arch of C1. This extended posteriorly around the right lateral side of the odontoid peg. There was also some minor bony spurring along the superior margin of the anterior C1 articular facet but no bony destruction.

Diagnostic CT reported a 15 x 12 mm focal nodular soft tissue thickening between the superior margin of the anterior arch of C1 and the clivus, with extension posteriorly around the right lateral side of the odontoid peg. There was also some minor bony spurring along the superior margin of the anterior C1 articular facet but no bony destruction. See Fig. 2.

The differential diagnosis at this point included joint-based pathology such as crystal deposition disease, metastatic cervical cancer, and a primary soft tissue tumor.

**Fig. 3:**
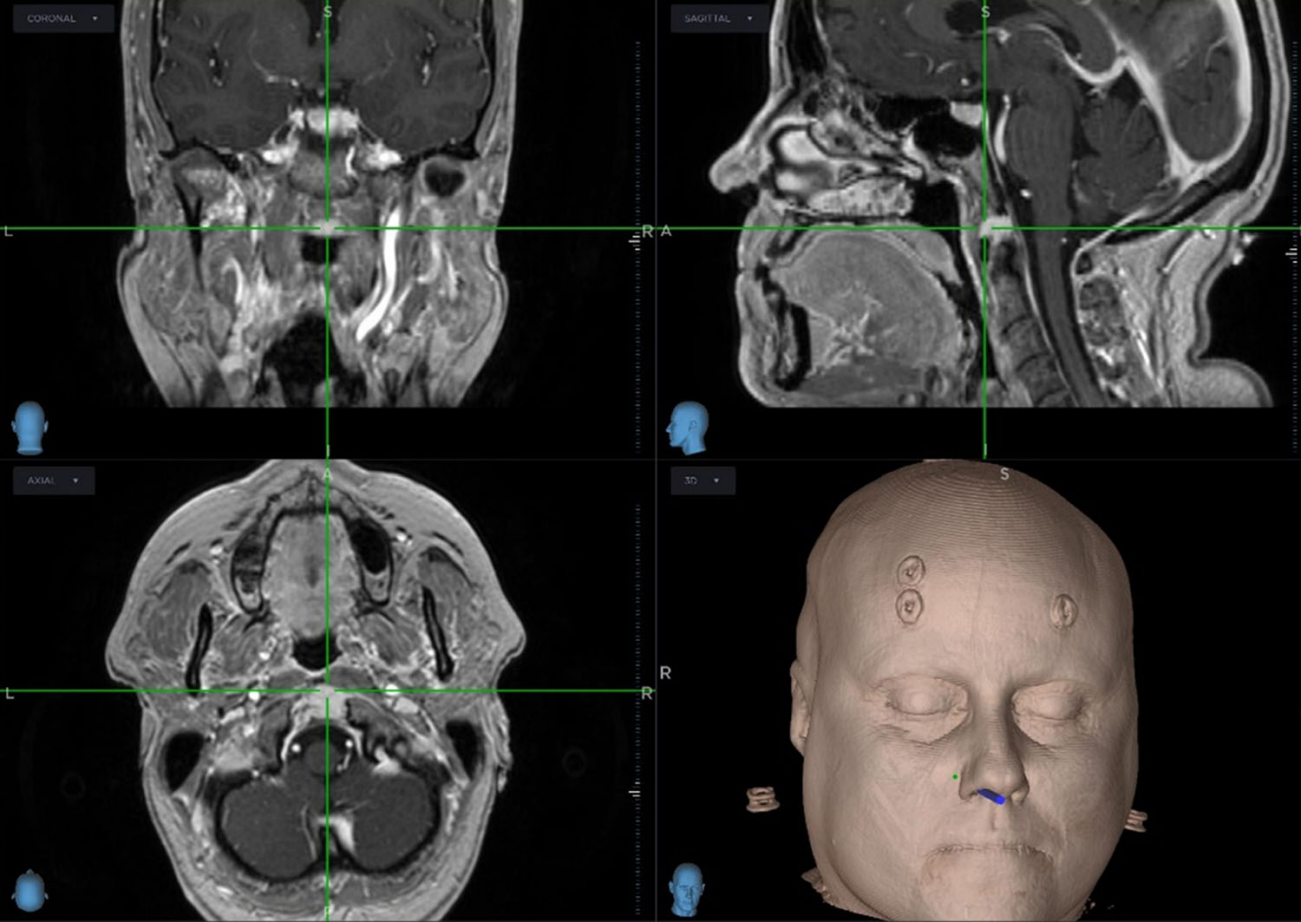
Case 1 stereotactic neuronavigational MRI. A soft tissue lesion was demonstrated and extended between the C1 and C2 articulation to the clivus. This was bound anteriorly by the anterior longitudinal ligament and posteriorly by the tectorial membrane. This measured 1.8 × 1.3 × 0.8 cm. The lesion was T2 hypointense and T1 isointense to muscle and did not demonstrate any diffusion restriction.

Repeated magnetic resonance imaging (MRI) showed a stable 16 x 10 mm uniformly enhancing mass on the superior surface of the anterior arch of C1, abutting the tip of the odontoid peg. There were no bony erosive changes to the odontoid peg, and it did not invade the prevertebral muscles at C1. The lesion was bound anteriorly by the anterior longitudinal ligament and posteriorly by the tectorial membrane. There was no compression of the spinal cord or central canal stenosis. The lesion was isointense on T1, hypotense on T2 and did not demonstrate any diffusion restriction. See Fig. 3.

Given its location, the lesion was biopsied surgically via a stereotactic endoscopic endonasal approach with otorhinolaryngologist support. After an inferiorly based flap was made in the nasopharynx, stereotaxis was used to identify the basion and the upper border of the C1 arch. The lesion was found in the space between the basion and the C1 arch and biopsied. It was hard, fibrous and had ill-defined margins.

Histology of the biopsy demonstrated a low grade fibrohistiocytic tumour and favoured the diagnosis of tenosynovial giant cell tumour. The specimen was of fibrotendinous connective tissue, with proliferation of mononuclear epithelioid to spindled cells within the fibrotic stroma. There was mild nuclear pleomorphism of these mononuclear cells. Giant cells were also seen. Both cells were positive for CD 68.

**Fig. 4:**
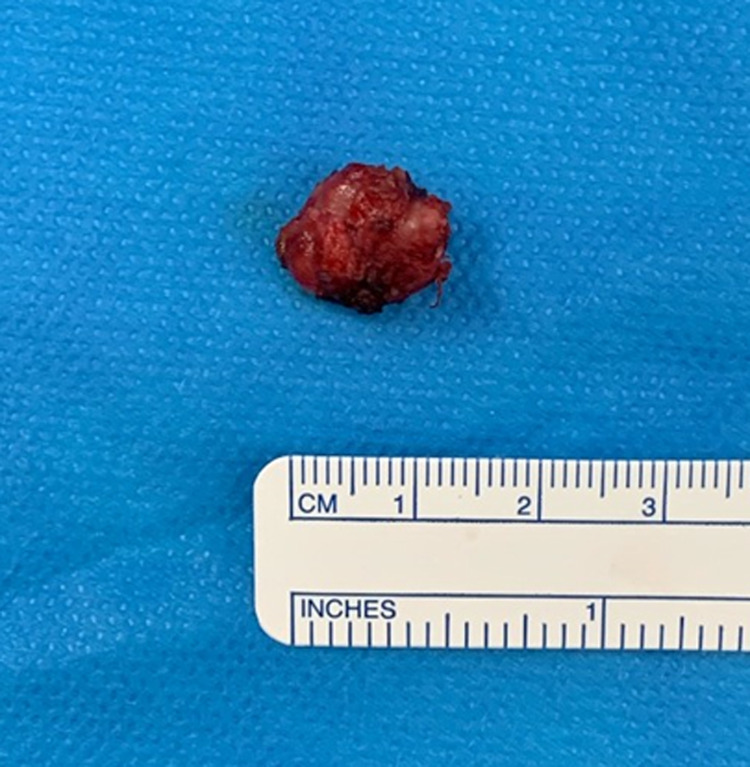
Resected C1/2 lesion.

Given the histological diagnosis, the patient underwent a gross total resection of the lesion. See Fig. 4. This was performed via a stereotactic transoral wide local excision. Using a Crockard transoral retractor system, the posterior pharyngeal wall and previous surgical incision were visualized. After stereotaxis was used to identify the C1 arch, the soft palate was divided along the midline and to the left of the uvula. The previous incision was opened and extended down to expose the C1 arch and the anterior rim of the foramen magnum. The tumour was found in the interspace between the rim and upper border of the C1 and dens, with extension along the space lateral to the tumour. Drilling out of the right side of the C1 arch provided adequate exposure for the tumour to be excised en bloc. Part of the apical and alar ligaments were removed due to infiltration by the tumor. There were no complications.

Post operatively, the patient reported instant relief of her neck pain and much improved neck and jaw range of movement. Patient also reported resolution of her proximal upper limb weakness, improved posture, and a more linear gait.

**Fig. 5:**
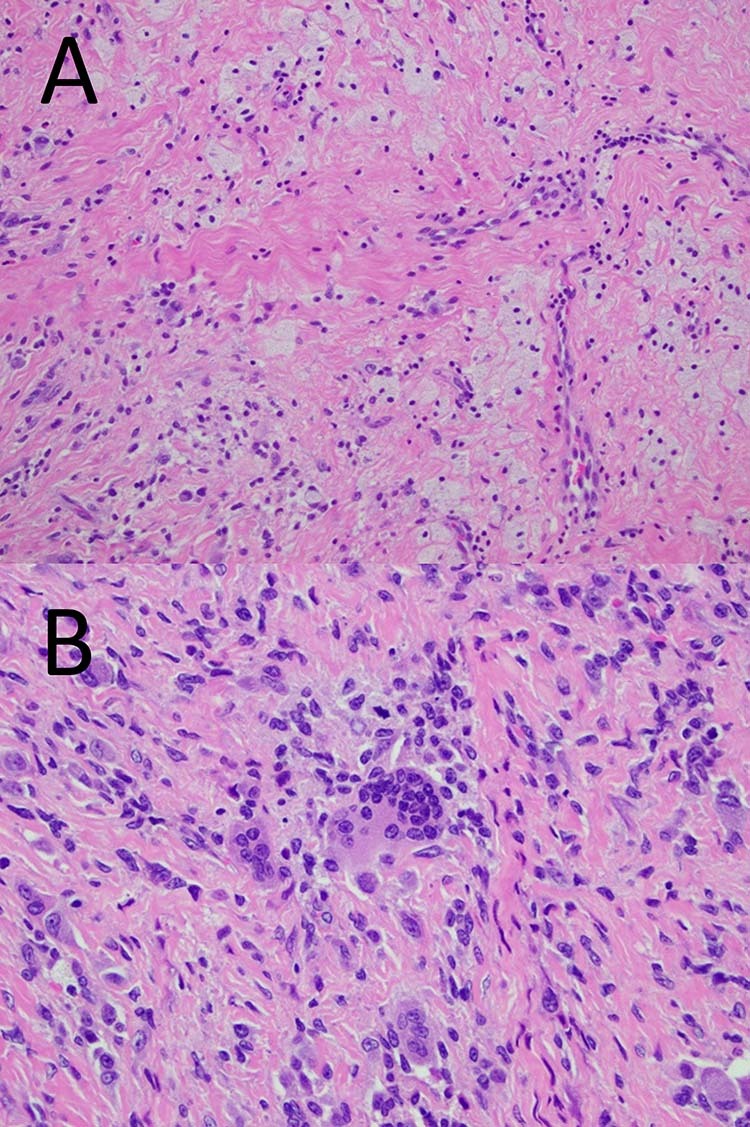
C1/2 lesion histology. **A** (Top) 200x image: The tumour was predominantly comprised of a mixture of mononuclear cells, foamy macrophages, and fibrosis. **B** (Bottom) 400x image: Focally multinucleated giant cells were also present (centre), where the nuclei had a similar appearance to the nuclei of background mononuclear cells.

Histology of the gross resection was in keeping with a localized tenosynovial giant cell tumour, with mononuclear cells positive for CD68 and factor XIIIa. The 25 × 25 × 6 mm circumscribed fibrohistiocytic lesion was surrounded by a fibrous pseudo-capsule and divided into lobules by fine septa. It was morphologically heterogenous, moderately cellular and contained abundant mononuclear cells, multi-multinucleated giant cells and foamy macrophages. There was focal haemosiderin deposition, rare inflammatory cells, and no crystal deposition. Tests for AE1/AE3, S100, p63, CD1a and BRAF V600E were all negative. See Fig. 5A, B.

Follow-up of the patient at 6 months showed complete resolution of symptoms. MRI of the cervical spine immediately post op and at 6 months excluded any residual or recurrence.

### Case 2 – C6/7 diffuse type TSGCT

A 48-year-old woman presented with an 11-month history of severe right sided headaches and an associated ‘heavy head’ sensation. She also reported neck pain with radiation into her right upper limb as well as severe vertigo. She had no change in limb power or sensation. Her symptoms progressed over 11 months and were transiently exacerbated each month during her menstrual cycle.

**Fig. 6:**
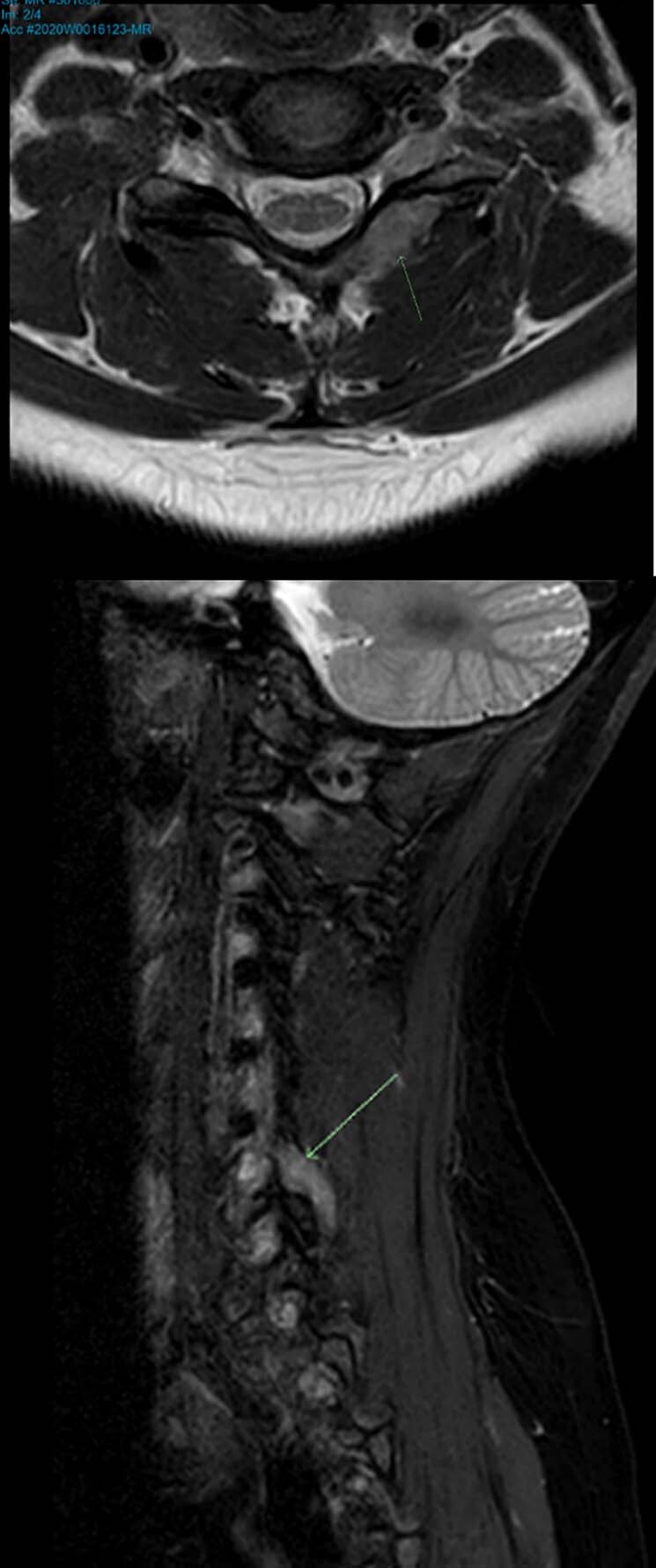
Case 2 MRI scan. There was a 19 mm intermediate T1/T2 signal area in the left posterior perivertebral tissues between the C6 and C7 lamina. This extended to the margin of the left facet joint.

She underwent an MRI of her brain and cervical spine to investigate the cause of her headache. MRI demonstrated a 19 mm intermediate T1/2 signal area in the left posterior perivertebral tissues between the C6 and C7 lamina, with extension to the margin of the left facet joint. It slightly expanded the left C6-7 facet and scalloped the left lamina of C7 without bone signal abnormality. The lesion was thought to be an incidental bone lesion rather than the cause of her symptoms and a CT scan was suggested to assess bone detail. See Fig. 6.

**Fig. 7:**
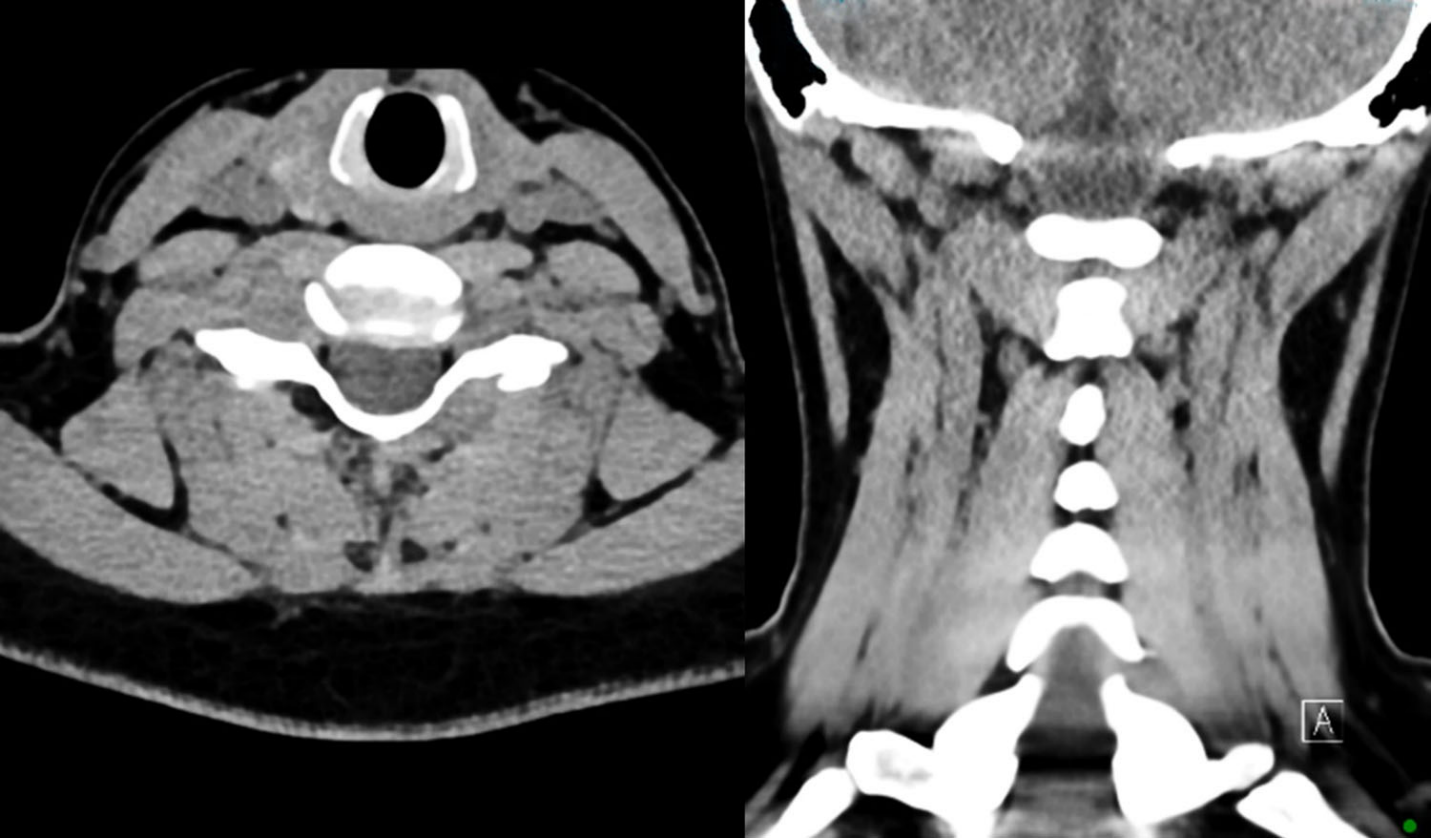
Case 2 CT scan. There was a soft tissue density focus at the left C7 lamina that extended to the medial aspect of the left C6/7 facet joint and measured 2 × 0.5 × 1.6 cm. There was bony remodeling of the left C6/7 articular column without overtly aggressive features.

Subsequent CT cervical spine excluded a bony lesion and advised of a soft tissue density focus (20x5x16mm). There was bony remodeling of the superior aspect of the left C7 articular column as well as an absence of cortical bone and the presence of a well-defined defect from the inferior aspect of the left C6 articular column. See Fig. 7.

Differential diagnoses at this point included a neuroma or schwannoma from a branch of the C7 dorsal root ganglion or a facet cyst with complex fluid content.

As the etiology was unclear at this point, the patient was referred for a CT guided core biopsy for further investigation. Histology of the core biopsy advised of a fibrohistiocytic tumour with a likely diagnosis of tenosynovial giant cell tumour. There was hypercellular proliferation of histiocytic cells and lobules of foamy macrophages associated with a fibrotic stroma. Mononuclear cells, focal multinucleate giant cells, foamy macrophages and siderophages were present. Majority of these cells were positive for CD68, with some occasional cells also showing p63 positivity.

**Fig. 8:**
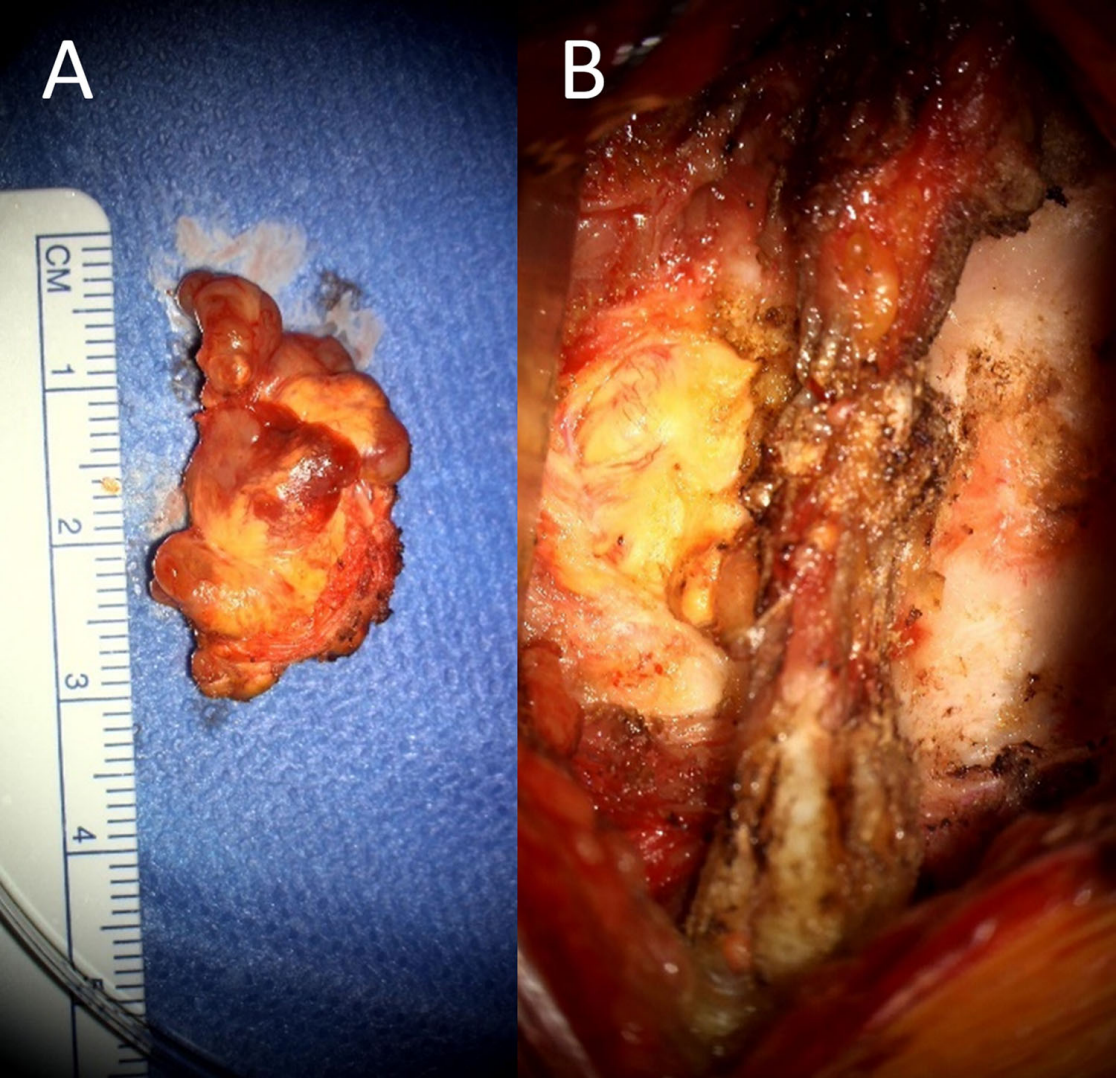
C6/7 lesion. **A** (Left) Resected C6/7 lesion. **B** (Right) Surgical photo of lesion insitu between the left C6 and C7 lamina.

Given the severity of the patient’s symptoms, she underwent a left sided C6/7 laminectomy and resection of the lesion. Intraoperatively, a discrete vascular lipomatous tumour with capsular delineation was found. It arose between the C6/7 lamina and followed the left C7 nerve into the foramen. The tumour was removed en bloc from the scalloped C7 lamina after undercutting of the C6 overlapping laminar. Small islands of tumour were also seen on dura and removed. The proximal facet joint was opened and debrided. Bone around the lamina margins was debrided by 3 mm. There were no complications. See Fig. 8A, B.

**Fig. 9:**
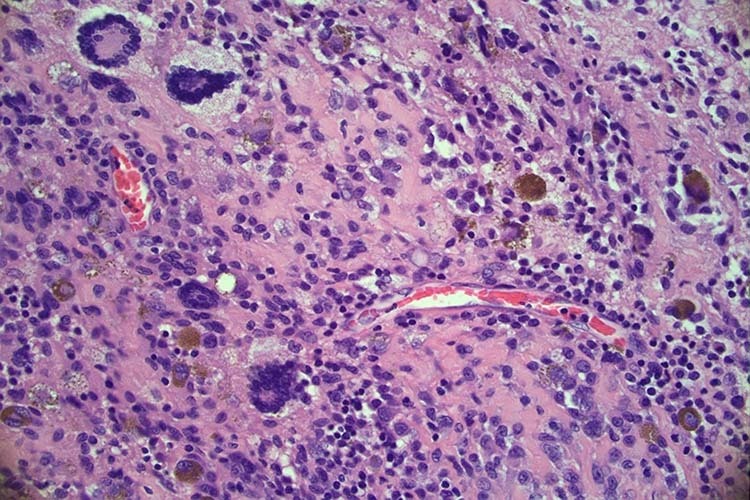
C6/7 histology. 40x image - characteristic diagnostic features of TSGCT – multinucleate giant cells, foamy histiocytes with pigment and bland spindled cells.

Histology of the gross resection demonstrated a diffuse type tenosynovial giant cell tumour. The lesion was described as an expansile nodular tumour with focal infiltration of fat and skeletal muscle. The tumour was hypercellular and composed of mononuclear cells, larger epithelioid cells, scatted osteoclast-like giant cells and sheets of foam cells with stromal hemosiderin deposition. See Fig. 9.

Cytogenetic analysis showed recurrent nonrandom translocation between the short arm of chromosome 1 and the long arm of chromosome 2 [t(1;2) (p13;q37)]. This fused CSF1 (1p13) to COL6A3, which was consistent with the diagnosis of TSGCT.

The patient’s symptoms significantly improved post operatively and were resolved within 6 weeks. Post operative MRI at 4 months excluded any residual or recurrent tumour.

## Discussion

The current literature regarding cervical TSGCTs, particularly those of the upper cervical spine, is limited. We discuss the unique location, new tissue biopsy method and surgical approach of our upper cervical lesion compared to our lower cervical lesion as well as the gold standard for investigation and management to improve understanding, diagnosis, and management of future cases.

### Location

TSGCTs usually arise from the synovium of tendon sheaths, bursa, or joints [2]. Large load bearing joints, such as the hips and knees, are most involved [1, 3]. Involvement of the axial skeleton is rare, with only ~80 cases having been reported in the literature [1, 8]. When present in the spine, 52% of lesions are found in the cervical spine while 29% of lesions occur in the lumbar spine and the remaining 17% of lesions are found in the thoracic spine [5]. Within the cervical spine, lesions of the upper cervical spine (C1/2) are extremely rare, with only 13 cases reported in the literature [2]. See Table 1. We report on one new case at C1/2 and another at C6/7.

**Table 1:** Known Upper Cervical (C1/2) Tenosynovial Giant Cell Tumours In the Literature.

Case	Author	Age	Symptoms	Site	Origin	Treatment
1	Pulitzer and Reed [9]	35 F	Neck pain	Paravertebral, hypopharyngeal	Synovial membranes of vertebral column accessory joint	Gross total resection
2	Graham, Kuklo, Kyriako, Rubin and Riew [10]	44 F	Neck pain	Right C1 + C2 lateral masses	Facet	Radiation, Gross total resection
3	Finn, McCall and Schmidt [11]	82 F	Neck pain, bilateral hand numbness, quadriparesis	Dens and right C2 lateral mass	Atlantoaxial joint	Radiological surveillance
4	Blankenbaker, Tuite, Koplin, Salamat and Hafez [6]	43 M	Asymptomatic	Posterior C1 arch	Bursa	Gross total resection
5	Teixeira, Lara, Narazaki, de Oliveira, Cavalcanti, Marins, et al. [7]	31 F	Neck pain	Right C1/2 intervertebral foramina	Facet	Gross total resection
6	Lavrador, Oliveira, Gil, Francisco and Livraghi [12]	64 M	Neck pain	Right C1 lateral mass	Facet	Radiological surveillance
7	Wang, Zhu, Yang, Liu, Yu and Liu [3]	23 F	Neck pain	R C1 lateral mass + C2 vertebral body	Facet	Gross total resection
8	Wang, Zhu, Yang, Liu, Yu and Liu [3]	44 F	Neck pain, reduced range of movement	Left C1/2 lateral masses and C2 vertebral body	Facet	Gross total resection
9	Yamada, Oshima, Hamada, Sotobori, Joyama, Hashimoto, et al. [13]	63 F	Asymptomatic	Vertebral membrane surrounding posterior arch of C1	Vertebral membrane	Gross total resection
10	Koontz, Quigley, Witt, Sanders and Shah [14]	49 F	Neck stiffness, jaw pain and headache	Dens, anterior arch + C1/2 right lateral masses	Atlantoaxial joint	Immunotherapy (imatinib)
11	Furuhata, Iwanami, Tsuji, Nagoshi, Suzuki, Okada, et al. [8]	32 F	Neck pain, myelopathy	Left C1/2 epidural space	Bursa, vertebral membrane	Gross total resection
12	Tsui, Fung, Chan, Yuen and Kan [5]	13 F	Asymptomatic	Left atlantoaxial joint	Facet	Radiological surveillance
13	Kim, Hong, Park, and Cho [2]	22 F	Palpable mass	Posterior atlanto-occipital membrane	Vertebral membrane	Gross total resection
Our case	Zhu, Campbell & Sadasivan	48 F	Neck pain + stiffness, UL weakness	Interspace between the rim and upper border of the C1 and dens	Atlantoaxial joint	Gross total resection

### Lesions with similar locations

Out of the 13 previously known cases of upper cervical spine (C1/2), only Koontz, Quigley [14] and Finn, McCall [11] reported lesions that were anterior to the dens. In Koontz, Quigley [14] the lesion was anterior to the dens and the right lateral mass. A trans-facial CT guided biopsy was performed for diagnosis. However, the lesion was deemed inoperable, and the patient was treated with immunotherapy, in the form of imatinib mesylate.

In Finn, McCall [11], the lesion infiltrated the atlas and the dens. Due to intervertebral instability, the patient underwent surgical stabilization using trans-articular screws and a posterior Dickman-Sonntag construct. During fixation of the cervical spine, the limited surgical access to the lesion from the posterior approach prevented gross total resection. They instead took a surgical biopsy for histological diagnosis. As the lesion was not able to be surgically resected, the patient was managed conservatively with radiological surveillance.

### Investigation and management

The investigation of cervical lesions begins with radiological imaging in the form of x-ray, CT, and MRI. X-rays can show erosive bone changes, destruction of the posterior elements of the vertebral body and occasionally a definable soft tissue mass. CT generally shows a hyperdense homogenously enhancing soft tissue mass with expansile osteolytic bone destruction and incomplete or thinning of cortical bone [1, 3, 12]. On MRI, lesions usually demonstrate hypointense or isointense signals on T1 and moderately hypointense signal on T2 [1]. In some cases, there is a mixed signal intensity on T2 weighted images due to the presence of hemosiderin, liquid, lipids, fibrous tissue, and hemorrhage [12, 13].

Imaging often lacks specific features for diagnosis [5]. There is a long list of differential diagnoses for cervical spinal masses, including aneurysmal bone cysts, granulomatous infection, synovial chrondromatosis, rheumatoid arthritis, osteoblastoma, gout, calcium pyrophosphate deposition disease, amyloid arthropathy, inflammatory arthropathy, and metastases [5, 6, 10]. Often, TSGCT is not considered due to its rarity. Subsequently, a surgical or CT guided biopsy of the lesion is usually arranged for histological diagnosis.

In general, gross total resection is recommended for TSGCTs due to their locally aggressive nature and the considerable risk for recurrence (20%) [1, 5–7]. In recurrent cases or those which are deemed inoperable, immunotherapy/chemotherapy, radiation therapy and radiological surveillance are used [2, 4, 8].

### Our cases (C1/2 and C6/7)

Both of our cases presented with typical symptoms of neck and radicular pain. Initial investigation of both cases was like previously documented cases, with CT and MRI imaging.

The uniqueness of our first case was the lesion’s anatomical location, our tissue biopsy method, and our surgical approach. Like the cases in Koontz, Quigley [14] and Finn, McCall [11], our lesion was located anterior to the dens. However, the proximity of our lesion to the posterior pharyngeal wall enabled surgical access to the lesion via either an endonasal or transoral approach with otorhinolaryngological support.

The initial biopsy of our lesion occurred by a stereotactic endoscopic endonasal approach (EEA), rather than a CT guided biopsy. EEA is a minimally invasive technique that was first introduced by Kassam, Snyderman [15] in 2005 for resection of the odontoid process [16]. It has a significantly lower rate of complications compared to a traditional transoral approach and is usually indicated for unstable C1/2 traumatic fractures, metastatic spine tumours and skull base osteomyelitis [17]. Our case is the first to utilize this tissue biopsy method for spinal TSGCTs.

For gross total resection of the lesion, we used a stereotactic transoral approach due to the lesion’s size (16 × 10 mm). The transoral approach is extensively described for pathologies of the anterior craniocervical junction [18]. However, this is the first case, to our knowledge, to use this technique for surgical resection of a cervical TSGCT. After reviewing radiological imaging from the other cases of anterior upper cervical TSCTs [11, 14], it is possible that a transoral approach could have been employed in these cases to facilitate gross total resection, rather than each case being deemed inoperable.

Our first case is juxtaposed with the more typical location of our second case (C6/7), where the lesion was in the left posterior perivertebral tissues between the C6 and C7 lamina. This more typical location allowed for a CT guided needle biopsy to be performed, followed by gross resection via a C6/7 laminectomy.

## Conclusion

TSGCTs are rare primary spinal tumours. They are often not considered as a differential diagnosis for soft tissues masses in the spine. Misdiagnosis is common and can have serious implications due to its locally aggressive nature and potential to cause joint instability and neurological deficits [2]. The unique location of our C1/2 lesion allowed for an endonasal biopsy and a gross total resection via a transoral approach.

We present two cases to hopefully improve knowledge of TSGCTs, to suggest more frequent inclusion of TSGCT as a differential diagnosis and to highlight our unique approach for biopsy and resection of anterior upper cervical lesions.

## Supplementary information

CARE CHECKLIST

## Data Availability

There was no data generated during this study. The histology images and results were provided by Mater Pathology. The radiological scans and reports are available from Queensland X-Ray, QScan and iMed Radiology.
