# Supplementary material for: Tenosynovial giant cell tumours of the upper and lower cervical spine: two case reports
Source: Spinal Cord Ser Cases. 2022 Aug 3;8:72. doi: 10.1038/s41394-022-00538-2 (PMC9349175; doi:10.1038/s41394-022-00538-2)
Supplement: Supplementary file 1 — CARE CHECKLIST [file 41394_2022_538_MOESM1_ESM.pdf]

CARE case report guidelines

CARE Checklist of information to include when writing a case report

(cc) BY-NC-ND

Topic	Item	Checklist item description	Reported on Line
Title	1	The diagnosis or intervention of primary focus followed by the words "case report"	2-3
Key Words	2	2 to 5 key words that identify diagnoses or interventions in this case report, including "case report"	37
Abstract (no references)	3a	Introduction: What is unique about this case and what does it add to the scientific literature?	24-28
	3b	Main symptoms and/or important clinical findings	30-31
	3c	The main diagnoses, therapeutic interventions, and outcomes	33-35
	3d	Conclusion—What is the main "take-away" lesson(s) from this case?	33-34
Introduction	4	One or two paragraphs summarizing why this case is unique (**may include references**)	44-62
Patient Information	5a	De-identified patient specific information.	69, 121
	5b	Primary concerns and symptoms of the patient.	69-71, 121-125
	5c	Medical, family, and psycho-social history including relevant genetic information	123-125
	5d	Relevant past interventions with outcomes	69, 121-125
Clinical Findings	6	Describe significant physical examination (PE) and important clinical findings.	70-71, 123
Timeline	7	Historical and current information from this episode of care organized as a timeline	69, 121-125
Diagnostic Assessment	8a	Diagnostic testing (such as PE, laboratory testing, imaging, surveys).	71-117, 126-159
	8b	Diagnostic challenges (such as access to testing, financial, or cultural)	79-80, 136-138
	8c	Diagnosis (including other diagnoses considered)	111, 152
	8d	Prognosis (such as staging in oncology) where applicable	118-119, 160-161
Therapeutic Intervention	9a	Types of therapeutic intervention (such as pharmacologic, surgical, preventive, self-care)	98-107, 145-151
	9b	Administration of therapeutic intervention (such as dosage, strength, duration)	98-107, 145-151
	9c	Changes in therapeutic intervention (with rationale)	98-107, 145-151
Follow-up and Outcomes	10a	Clinician and patient-assessed outcomes (if available)	118-119, 160-161
	10b	Important follow-up diagnostic and other test results	119, 161
	10c	Intervention adherence and tolerability (How was this assessed?)	108-110, 118, 160
	10d	Adverse and unanticipated events	107, 151
Discussion	11a	A scientific discussion of the strengths AND limitations associated with this case report	210-234
	11b	Discussion of the relevant medical literature **with references**.	169-206
	11c	The scientific rationale for any conclusions (including assessment of possible causes)	212-230
	11d	The primary "take-away" lessons of this case report (without references) in a one paragraph conclusion	237-244
Patient Perspective	12	The patient should share their perspective in one to two paragraphs on the treatment(s) they received	108-110, 160
Informed Consent	13	Did the patient give informed consent? Please provide if requested	Yes ☒ No ☐
